# Supplementary material for: Effects of Different Nitrogen Levels on Lignocellulolytic Enzyme Production and Gene Expression under Straw-State Cultivation in Stropharia rugosoannulata
Source: Int J Mol Sci. 2023 Jun 13;24(12):10089. doi: 10.3390/ijms241210089 (PMC10298262; doi:10.3390/ijms241210089)
Supplement: Supplementary file 1 [file ijms-24-10089-s001.zip › Supplemental Material.pdf]

Supplementary Figure Captions

Figure S1 GO and KEGG enrichment of the 36 DEGs. The top 20 GO (A) and KEGG (B) with the pvalue, DEGs number, name and rich_factor.

Figure S2 The GO enrichment of the DEGs. The top 20 GO with the FDR, DEGs number and GO name between A1 vs. A2 (A) and A2 vs. A3 (B). The degree of enrichment was indicated by the rich factor, FDR values, and number of genes enriched in the pathway. A larger rich factor indicates a greater degree of enrichment. The FDR generally ranges from 0-1, and a value closer to zero indicates, more significant enrichment. The top 20 KEGG pathways with the smallest FDR values, namely, the most significant enrichment was selected for display

Figure S3 Differential expression of carbohydrate enzyme genes at different nitrogen levels. (A) Differential expression of AA genes. (B) Differential expression of GH genes. (C) Differential expression of CE genes. (D) Differential expression of CBM genes. (E) Differential expression of GT genes and (F) Differential expression of PL genes.

Supplementary Tables

Supplementary Table S1 Primers for qRT-PCR of the validation gene

Gene Name	Gene ID	Primer-F	Primer-R
AA9	DQGG009829	CTGGACCTCCACCATCCCTA	GAGCTCAGCTGAAGTGGGTT
AA10	DQGG003510	GACGCGAACTTTGTGGATCA	TGTCGATCGAAGTGAGGAGG
GH5	DQGG009707	ACTCCTACAGCCAGTCAACC	CCAAGACAGGCTCCATCAGA
GH10	DQGG010271	CTCTCTTTCGTCCATGCAGC	TTGATGTCAGCGAGAAGGGT
CBM1	DQGG006793	AAGTGTCGTGAAGCTCGGTA	ACAAGCCACGCATTAGTCTG

GT2	DQGG007143	CCACAAAGAGAAACCACGCA	CGAAGCGTGTTGTACTACCG
GT8	DQGG005901	TCTGGAGCAGGACAACGCT	CGGGTACAGCAGAAAATTCG
CE16	DQGG004257	ATCCTCCCTCCCCATCTCTT	TGGCCCGTGGTGTTATAGTT
PLY14	DQGG004094	ACTTGAGCGACTGGTGGTTA	TCCCGACTTTCCTTTACCCC
GOGAT	DQGG006969	GAATGGACGAAGGATAGCGGA	AAGAAGCCCAGAGCCAAGAAA
AS	DQGG006918	AAAGCAATGGGAGTCAA	ACGAAGGCAGTCAGAAG
GS	DQGG001244	TACCCATACCGCAACGAC	CGAACGGAGACCACCATC
GDH	DQGG009462	GCAAGTCCGACAACGAGA	CTGTAAGCACCGAAGAGG
NR	DQGG006945	ATATGACCGAAAAGGTAGT	TCTGTGTTATTGGAGGAGT
NiR	DQGG001090	ACACGATGACATACCTAA	AAGAATGAAAACAAAAAA
Cx	DQGG008787	ACCTCTCTCGTTGCTTG	CTGGTGAATTGCTGACT
C1	DQGG008064	ACCCCTGCTTACTTCCCA	CCATCACATTCACCTCCG
β-GA	DQGG006934	GACTCCTCACCTTCCACCCCT	TTTCTTTCTTGGCCCCTTGTT
β-GC	DQGG009756	GGACTTTGCTATCGGTT	CGTGTACGTGTTCATGC
ACX	DQGG002951	GGGTTGAGGCAGGAAAT	CCGTCATCGTTAAAGGG
MAPK	DQGG006110	CTTTTCCGAAACCTGCGTCA	AGTACTGGCAATGGTCGTCA
PBA	DQGG001226	ATGTTGGTCATTTCGATCCT	TGCGGTACCAGCTCCGACGT
SHO1	DQGG006454	GAGTCACCATGGTCGAGGA	ACAAGGGATGGGACCGTG
18s	DQGG007047	TTGAACTTCGTGGAGTCGGG	TTAAACCGTACCCAGGCGAC

Supplementary Table S2 Summary of the sequencing data of *Stropharia rugosoannulata* transcriptome at three different nitrogen concentrations.

Sample	Reads No	Bases(bp)	Q30(bp)	N(%)	Q20(%)	Q30(%)	**Multiple_Mapped**	**Uniquely_Mapped**
A1-1	48250584	7237587600	6775184729	0.050839	97.55	93.61	11038228	29370596 (72.68%)

							(27.32%)	
A1-2	41665822	6249873300	5813606120	0.050663	97.12	93.01	769232 (2.18%)	34583996 (97.82%)
A1-3	41810872	6271630800	5879569701	0.050364	97.63	93.74	8790971 (24.78%)	26689682 (75.22%)
A2-1	40405832	6060874800	5669485916	0.050091	97.44	93.54	574047 (1.65%)	34268069 (98.35%)
A2-2	40840062	6126009300	5737015780	0.049803	97.52	93.65	3209033 (9.17%)	31767339 (90.83%)
A2-3	37415136	5612270400	5241727767	0.050447	97.37	93.39	1166920 (3.63%)	30935505 (96.37%)
A3-1	44926540	6738981000	6301950522	0.050469	97.48	93.51	777068 (1.99%)	38329234 (98.01%)
A3-2	42560976	6384146400	5985874295	0.048009	97.57	93.76	801239 (2.16%)	36235787 (97.84%)
A3-3	45262082	6789312300	6355435887	0.050325	97.48	93.6	695631 (1.77%)	38631547 (98.23%)

Supplementary Table S3 GO pathways enrichment analysis of differentially expressed genes.

A2 VS A3						
**Category**	**GO.ID**	**Term**	**DEG**	**Total**	**Pvalue**	**FDR**
BP	GO:0043605	cellular amide catabolic process	8	8	$1.12054E^{-07}$	0.000520493
BP	GO:0015821	methionine transport	10	13	$3.93081E^{-07}$	0.00091293
MF	GO:0015191	L-methionine transmembrane transporter activity	10	13	$4.81059E^{-07}$	0.000294168
MF	GO:0043865	methionine transmembrane transporter activity	10	13	$4.81059E^{-07}$	0.000294168
CC	GO:0005576	extracellular region	56	230	$8.66402E^{-07}$	0.000680126
BP	GO:0000101	sulfur amino acid transport	10	14	$1.20815E^{-06}$	0.001870618
MF	GO:0000099	sulfur amino acid transmembrane transporter activity	10	14	$1.47429E^{-06}$	0.000601017
BP	GO:1900034	regulation of cellular response to heat	15	33	$8.2988E^{-06}$	0.009636981
MF	GO:0004553	hydrolase activity, hydrolyzing O-glycosyl compounds	44	166	$9.58308E^{-06}$	0.002930026
BP	GO:0010410	hemicellulose metabolic process	15	36	$2.99242E^{-05}$	0.022751522
BP	GO:0000272	polysaccharide catabolic process	31	109	$3.11483E^{-05}$	0.022751522

BP	GO:0006805	xenobiotic metabolic process	9	15	$3.49104E^{-05}$	0.022751522
BP	GO:0016998	cell wall macromolecule catabolic process	15	37	$4.40826E^{-05}$	0.022751522
BP	GO:0044347	cell wall polysaccharide catabolic process	15	37	$4.40826E^{-05}$	0.022751522
MF	GO:0016798	hydrolase activity, acting on glycosyl bonds	45	184	$6.73182E^{-05}$	0.012265683
MF	GO:0008137	NADH dehydrogenase (ubiquinone) activity	10	19	$7.02042E^{-05}$	0.012265683
MF	GO:0050136	NADH dehydrogenase (quinone) activity	10	19	$7.02042E^{-05}$	0.012265683
BP	GO:0006022	aminoglycan metabolic process	16	43	$8.49581E^{-05}$	0.039463061
BP	GO:0042737	drug catabolic process	27	95	0.000100979	0.042640654
BP	GO:0071466	cellular response to xenobiotic stimulus	9	17	0.000131293	0.050821226
A3 VS A1						
**Category**	**GO.ID**	**Term**	**DEG**	**Total**	**Pvalue**	**FDR**
MF	GO:0015291	secondary active transmembrane transporter activity	100	259	$2.68544E^{-14}$	$3.77304E^{-11}$
MF	GO:0015297	antiporter activity	79	207	$3.18849E^{-11}$	$2.23992E^{-08}$
BP	GO:0098656	anion transmembrane transport	117	363	$4.91031E^{-11}$	$2.36924E^{-07}$
MF	GO:0008514	organic anion transmembrane transporter activity	99	285	$6.04523E^{-11}$	$2.83118E^{-08}$
BP	GO:0006855	drug transmembrane transport	91	263	$1.26217E^{-10}$	$2.36924E^{-07}$
MF	GO:0008509	anion transmembrane transporter activity	105	312	$1.31171E^{-10}$	$4.60737E^{-08}$
BP	GO:0015893	drug transport	98	291	$1.3536E^{-10}$	$2.36924E^{-07}$
BP	GO:0006820	anion transport	137	454	$1.84884E^{-10}$	$2.42706E^{-07}$
CC	GO:0005576	extracellular region	79	230	$7.14165E^{-10}$	$6.05612E^{-07}$
MF	GO:0022804	active transmembrane transporter activity	128	415	$8.0927E^{-10}$	$2.27405E^{-07}$
BP	GO:0015711	organic anion transport	127	421	$9.36676E^{-10}$	$9.83697E^{-07}$
MF	GO:0004553	hydrolase activity, hydrolyzing O-glycosyl compounds	64	166	$1.55863E^{-09}$	$3.64397E^{-07}$

BP	GO:0000272	polysaccharide catabolic process	47	109	$1.58378E^{-09}$	$1.38608E^{-06}$
MF	GO:0015301	anion:anion antiporter activity	64	167	$2.07486E^{-09}$	$3.64397E^{-07}$
MF	GO:0140323	solute:anion antiporter activity	64	167	$2.07486E^{-09}$	$3.64397E^{-07}$
BP	GO:0006811	ion transport	185	684	$2.47388E^{-09}$	$1.85576E^{-06}$
MF	GO:0016798	hydrolase activity, acting on glycosyl bonds	68	184	$3.85669E^{-09}$	$6.02073E^{-07}$
BP	GO:0072530	purine-containing compound transmembrane transport	63	171	$6.2278E^{-09}$	$4.08777E^{-06}$
BP	GO:0034220	ion transmembrane transport	157	566	$7.40271E^{-09}$	$4.25839E^{-06}$
MF	GO:0022857	transmembrane transporter activity	172	620	$7.96051E^{-09}$	$1.06791E^{-06}$
A1VS A2						
**Category**	**GO.ID**	**Term**	**DEG**	**Total**	**Pvalue**	**FDR**
MF	GO:0015291	secondary active transmembrane transporter activity	67	259	$9.82583E^{-13}$	$5.39831E^{-09}$
BP	GO:0006855	drug transmembrane transport	66	263	$5.03074E^{-12}$	$9.19734E^{-09}$
BP	GO:1901679	nucleotide transmembrane transport	50	172	$7.0244E^{-12}$	$9.19734E^{-09}$
MF	GO:0015215	nucleotide transmembrane transporter activity	50	172	$8.96664E^{-12}$	$9.19734E^{-09}$
MF	GO:0015301	anion:anion antiporter activity	49	167	$1.00444E^{-11}$	$9.19734E^{-09}$
MF	GO:0140323	solute:anion antiporter activity	49	167	$1.00444E^{-11}$	$9.19734E^{-09}$
MF	GO:0015932	nucleobase-containing compound transmembrane transporter activity	52	186	$1.74633E^{-11}$	$1.12509E^{-08}$
MF	GO:0015605	organophosphate ester transmembrane transporter activity	51	181	$2.00438E^{-11}$	$1.12509E^{-08}$
BP	GO:0140021	mitochondrial ADP transmembrane transport	47	160	$2.04786E^{-11}$	$1.12509E^{-08}$
BP	GO:1990544	mitochondrial ATP transmembrane transport	47	160	$2.04786E^{-11}$	$1.12509E^{-08}$
MF	GO:0005347	ATP transmembrane transporter activity	47	160	$2.59107E^{-11}$	$1.14798E^{-08}$

MF	GO:0005471	ATP:ADP antiporter activity	47	160	$2.59107E^{-11}$	$1.14798E^{-08}$
BP	GO:0015893	drug transport	69	291	$2.71637E^{-11}$	$1.14798E^{-08}$
BP	GO:0015866	ADP transport	47	162	$3.31591E^{-11}$	$1.30126E^{-08}$
MF	GO:0015216	purine nucleotide transmembrane transporter activity	48	167	$3.71882E^{-11}$	$1.35458E^{-08}$
BP	GO:0006862	nucleotide transport	52	191	$4.07668E^{-11}$	$1.35458E^{-08}$
MF	GO:0015217	ADP transmembrane transporter activity	47	162	$4.19146E^{-11}$	$1.35458E^{-08}$
BP	GO:0072530	purine-containing compound transmembrane transport	48	171	$7.36362E^{-11}$	$2.17329E^{-08}$
MF	GO:1901505	carbohydrate derivative transmembrane transporter activity	51	187	$7.51594E^{-11}$	$2.17329E^{-08}$
MF	GO:0000295	adenine nucleotide transmembrane transporter activity	47	166	$1.06456E^{-10}$	$2.7851E^{-08}$

Supplementary Table S4 KEGG pathways enrichment analysis of differentially expressed genes.

A2 VS A3							
Pathway ID	Pathway	Level1	Level2	DEG_number	total_number	Pvalue	FDR
ko03030	DNA replication	Genetic Information Processing	Replication and repair	17	38	$9.4071E^{-09}$	$8.0901E^{-07}$
ko00330	Arginine and proline metabolism	Metabolism	Amino acid metabolism	12	27	$1.79501E^{-06}$	$7.71855E^{-05}$

ko03430	Mismatch repair	Genetic Information Processing	Replication and repair	10	24	$2.76271E^{-05}$	0.000791976
ko03410	Base excision repair	Genetic Information Processing	Replication and repair	9	25	0.000270848	0.005823238
ko03420	Nucleotide excision repair	Genetic Information Processing	Replication and repair	11	39	0.000641837	0.00932866
ko00910	Nitrogen metabolism	Metabolism	Energy metabolism	6	13	0.000650837	0.00932866
ko00053	Ascorbate and aldarate metabolism	Metabolism	Carbohydrate metabolism	6	14	0.001050149	0.011907522
ko00360	Phenylalanine metabolism	Metabolism	Amino acid metabolism	7	19	0.001150592	0.011907522
ko00380	Tryptophan metabolism	Metabolism	Amino acid metabolism	9	30	0.001246136	0.011907522
ko00260	Glycine, serine and threonine metabolism	Metabolism	Amino acid metabolism	12	52	0.00252509	0.021715776
ko03440	Homologous recombination	Genetic Information Processing	Replication and repair	7	22	0.003043616	0.023795546
ko03450	Non-homologous end-joining	Genetic Information Processing	Replication and repair	4	10	0.010387896	0.074446588
ko00500	Starch and sucrose metabolism	Metabolism	Carbohydrate metabolism	11	55	0.011845689	0.078363787
ko00450	Selenocompound metabolism	Metabolism	Metabolism of other amino acids	3	7	0.022013185	0.135223852
ko00480	Glutathione metabolism	Metabolism	Metabolism of other amino acids	6	25	0.025595475	0.146747392

ko00740	Riboflavin metabolism	Metabolism	Metabolism of cofactors and vitamins	4	13	0.028203897	0.151595947
ko00520	Amino sugar and nucleotide sugar metabolism	Metabolism	Carbohydrate metabolism	10	56	0.034015207	0.172076929
ko00620	Pyruvate metabolism	Metabolism	Carbohydrate metabolism	8	44	0.050479735	0.241180957
ko00565	Ether lipid metabolism	Metabolism	Lipid metabolism	2	5	0.073783743	0.333968523
ko00232	Caffeine metabolism	Metabolism	Biosynthesis of other secondary metabolites	1	1	0.094804499	0.388246997
A3 VS A1							
**Pathway ID**	**Pathway**	**Level1**	**Level2**	**DEG_number**	**total_number**	**Pvalue**	**FDR**
ko00360	Phenylalanine metabolism	Metabolism	Amino acid metabolism	11	19	$3.59066E^{-06}$	0.000337523
ko00330	Arginine and proline metabolism	Metabolism	Amino acid metabolism	12	27	$4.38243E^{-05}$	0.00205974
ko00520	Amino sugar and nucleotide sugar metabolism	Metabolism	Carbohydrate metabolism	17	56	0.000358572	0.011235267
ko03030	DNA replication	Genetic Information Processing	Replication and repair	13	38	0.000503182	0.011824769

ko00630	Glyoxylate and dicarboxylate metabolism	Metabolism	Carbohydrate metabolism	9	25	0.002528896	0.038060299
ko04011	MAPK signaling pathway - yeast	Environmental Information Processing	Signal transduction	18	71	0.002531647	0.038060299
ko00380	Tryptophan metabolism	Metabolism	Amino acid metabolism	10	30	0.002834278	0.038060299
ko00910	Nitrogen metabolism	Metabolism	Energy metabolism	6	13	0.003245644	0.038136318
ko00260	Glycine, serine and threonine metabolism	Metabolism	Amino acid metabolism	14	52	0.004214642	0.044019599
ko00480	Glutathione metabolism	Metabolism	Metabolism of other amino acids	8	25	0.009823894	0.083949638
ko00350	Tyrosine metabolism	Metabolism	Amino acid metabolism	8	25	0.009823894	0.083949638
ko00052	Galactose metabolism	Metabolism	Carbohydrate metabolism	7	21	0.012247637	0.095939821
ko00620	Pyruvate metabolism	Metabolism	Carbohydrate metabolism	11	44	0.019105932	0.138150584
ko04111	Cell cycle - yeast	Cellular Processes	Cell growth and death	19	94	0.025014587	0.167955087
ko03450	Non-homologous end-joining	Genetic Information Processing	Replication and repair	4	10	0.029356254	0.178808822
ko00603	Glycosphingolipid biosynthesis - globo and isoglobo series	Metabolism	Glycan biosynthesis and metabolism	3	6	0.030827093	0.178808822

ko03410	Base excision repair	Genetic Information Processing	Replication and repair	7	25	0.032337766	0.178808822
ko00680	Methane metabolism	Metabolism	Energy metabolism	8	31	0.036250155	0.189306365
ko00500	Starch and sucrose metabolism	Metabolism	Carbohydrate metabolism	12	55	0.040638503	0.201053648
ko00130	Ubiquinone and other terpenoid-quinone biosynthesis	Metabolism	Metabolism of cofactors and vitamins	3	7	0.048928433	0.229963634
A1 VS A2							
**Pathway ID**	**Pathway**	**Level1**	**Level2**	**DEG_number**	**total_number**	**Pvalue**	**FDR**
ko00620	Pyruvate metabolism	Metabolism	Carbohydrate metabolism	9	44	0.005155192	0.205408778
ko00520	Amino sugar and nucleotide sugar metabolism	Metabolism	Carbohydrate metabolism	10	56	0.008875565	0.205408778
ko00300	Lysine biosynthesis	Metabolism	Amino acid metabolism	4	12	0.010182085	0.205408778
ko00040	Pentose and glucuronate interconversions	Metabolism	Carbohydrate metabolism	5	19	0.012082869	0.205408778
ko00260	Glycine, serine and threonine metabolism	Metabolism	Amino acid metabolism	9	52	0.015628173	0.212543146
ko00250	Alanine, aspartate and glutamate metabolism	Metabolism	Amino acid metabolism	6	30	0.023680647	0.268380666

ko03008	Ribosome biogenesis in eukaryotes	Genetic Information Processing	Translation	9	58	0.030306662	0.294407577
ko00052	Galactose metabolism	Metabolism	Carbohydrate metabolism	4	21	0.071597857	0.497124851
ko00910	Nitrogen metabolism	Metabolism	Energy metabolism	3	13	0.071641884	0.497124851
ko00680	Methane metabolism	Metabolism	Energy metabolism	5	31	0.083718794	0.497124851
ko00650	Butanoate metabolism	Metabolism	Carbohydrate metabolism	3	14	0.086208622	0.497124851
ko03020	RNA polymerase	Genetic Information Processing	Transcription	4	23	0.094108257	0.497124851
ko00450	Selenocompound metabolism	Metabolism	Metabolism of other amino acids	2	7	0.095038574	0.497124851
ko00630	Glyoxylate and dicarboxylate metabolism	Metabolism	Carbohydrate metabolism	4	25	0.119532405	0.568263453
ko00230	Purine metabolism	Metabolism	Nucleotide metabolism	5	35	0.125352232	0.568263453
ko00100	Steroid biosynthesis	Metabolism	Lipid metabolism	3	17	0.136328813	0.579397457
ko00470	D-Amino acid metabolism	Metabolism	Metabolism of other amino acids	1	2	0.147411695	0.589646779
ko00310	Lysine degradation	Metabolism	Amino acid metabolism	4	28	0.162455731	0.613721649

ko00030	Pentose phosphate pathway	Metabolism	Carbohydrate metabolism	3	22	0.235151059	0.825023641
ko00500	Starch and sucrose metabolism	Metabolism	Carbohydrate metabolism	6	55	0.242654012	0.825023641
