# Supplementary figures and images for: Effects of Different Nitrogen Levels on Lignocellulolytic Enzyme Production and Gene Expression under Straw-State Cultivation in Stropharia rugosoannulata
Source: Int J Mol Sci. 2023 Jun 13;24(12):10089. doi: 10.3390/ijms241210089 (PMC10298262; doi:10.3390/ijms241210089)

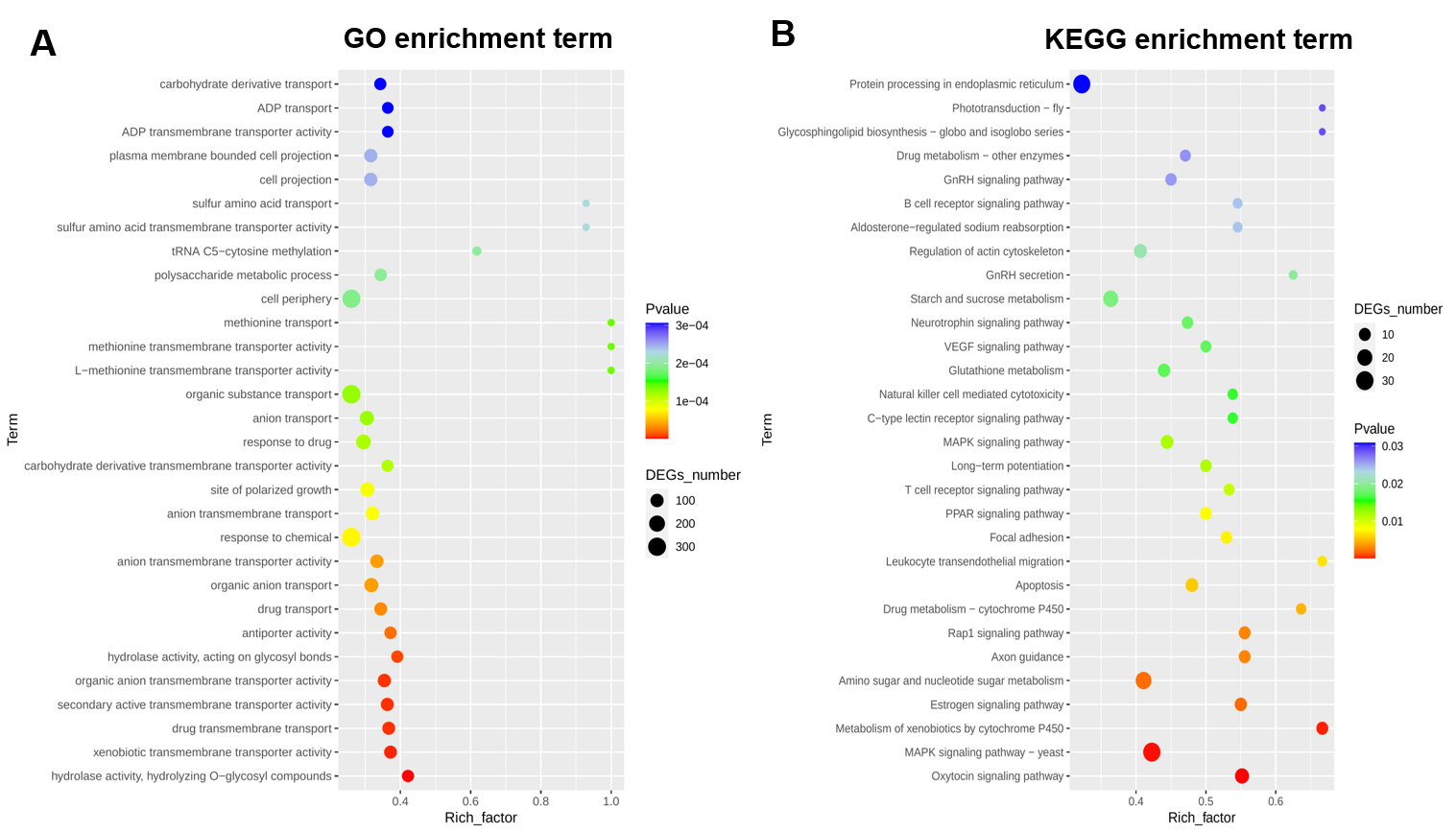

Supplement: Supplementary file 1 [file ijms-24-10089-s001.zip › Figure S1.tif]

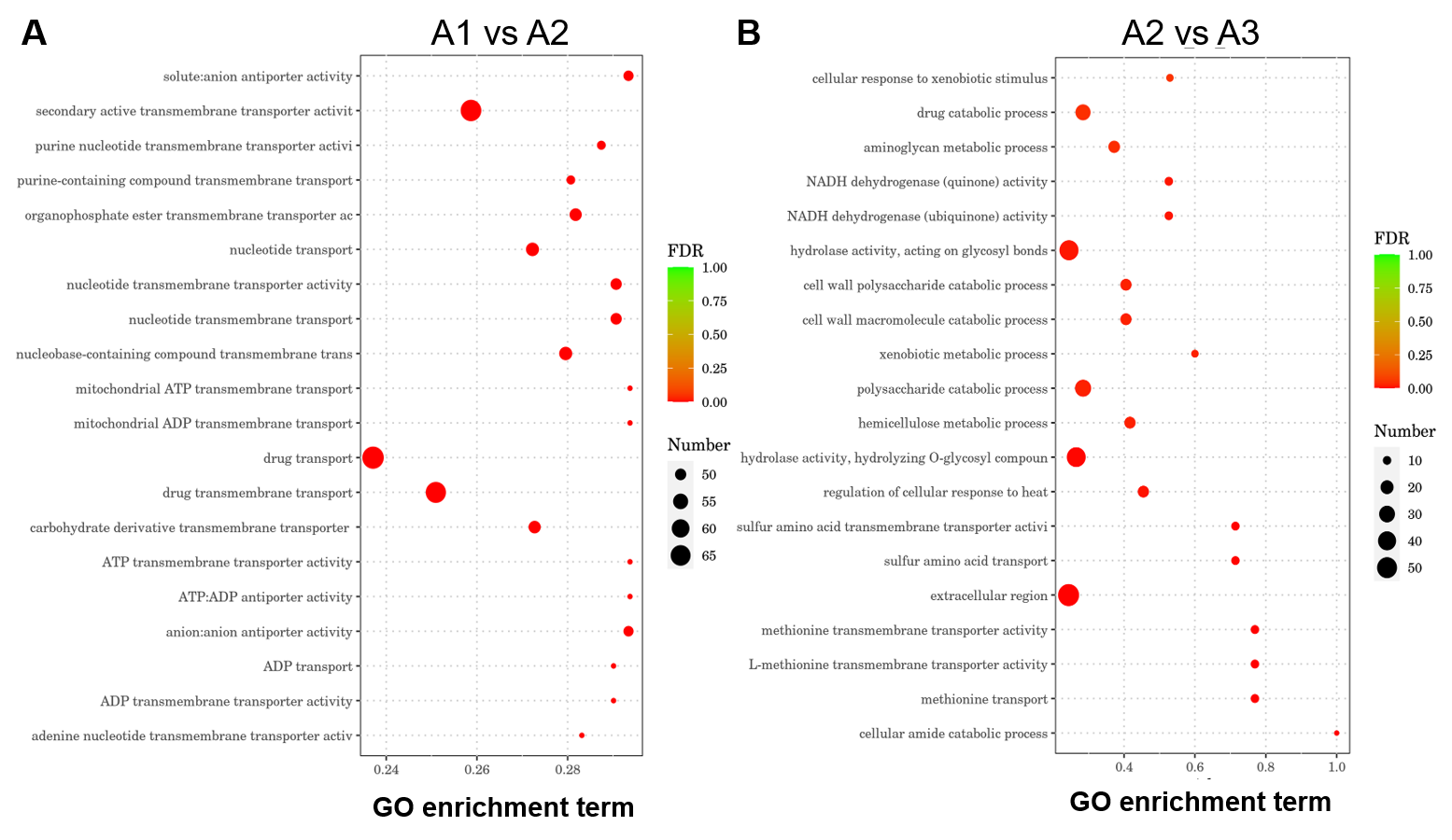

Supplement: Supplementary file 1 [file ijms-24-10089-s001.zip › Figure S2.tif]

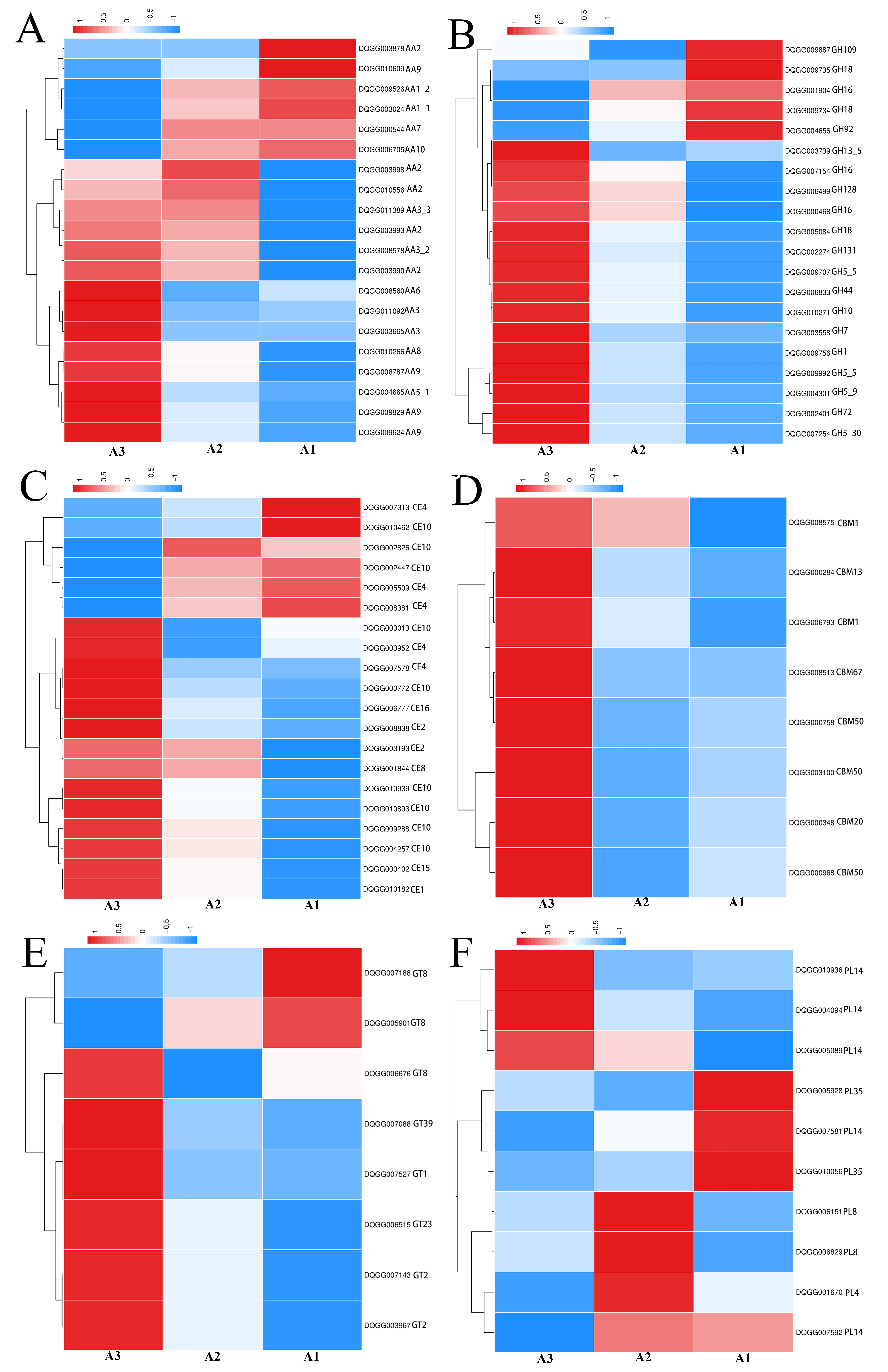

Supplement: Supplementary file 1 [file ijms-24-10089-s001.zip › Figure S3.tif]
